# HCV-specific CD4+ T cells of patients with acute and chronic HCV infection display high expression of TIGIT and other co-inhibitory molecules

**DOI:** 10.1038/s41598-019-47024-8

**Published:** 2019-07-23

**Authors:** Christin Ackermann, Maike Smits, Robin Woost, Johanna M. Eberhard, Sven Peine, Silke Kummer, Matthias Marget, Thomas Kuntzen, William W. Kwok, Ansgar W. Lohse, Thomas Jacobs, Tobias Boettler, Julian Schulze zur Wiesch

**Affiliations:** 1 I. Department of Medicine, University Medical Center Hamburg-Eppendorf, Hamburg, Germany; 2 Department of Medicine II, Medical Center-University of Freiburg, Faculty of Medicine, University of Freiburg, Freiburg, Germany; 3 Faculty of Biology, University of Freiburg, Freiburg, Germany; 4 DZIF partner site (German Center for Infection Research), Hamburg, Germany; 5 Department of Transfusion Medicine, Germany, University Medical Center Hamburg-Eppendorf, Hamburg, Germany; 6 Gastroenterologie und Hepatologie; Kantonsspital Aarau, Aarau, Switzerland; 7 Benaroya Research Institute at Virginia Mason, Seattle, Washington United States of America; 8 Protozoa Immunology, Bernhard Nocht, Institute for Tropical Medicine, Hamburg, Germany

**Keywords:** Hepatitis C virus, Immunological memory, Hepatitis C

## Abstract

The combined regulation of a network of inhibitory and activating T cell receptors may be a critical step in the development of chronic HCV infection. *Ex vivo* HCV MHC class I + II tetramer staining and bead-enrichment was performed with baseline and longitudinal PBMC samples of a cohort of patients with acute, chronic and spontaneously resolved HCV infection to assess the expression pattern of the co-inhibitory molecule TIGIT together with PD-1, BTLA, Tim-3, as well as OX40 and CD226 (DNAM-1) of HCV-specific CD4+ T cells, and in a subset of patients of HCV-specific CD8+ T cells. As the main result, we found a higher expression level of TIGIT+ PD-1+ on HCV-specific CD4+ T cells during acute and chronic HCV infection compared to patients with spontaneously resolved HCV infection (p < 0,0001). Conversely, expression of the complementary co-stimulatory receptor of TIGIT, CD226 (DNAM-1) was significantly decreased on HCV-specific CD4+ T cells during chronic infection. The predominant phenotype of HCV-specific CD4+ T cells during acute and chronic infection was TIGIT+, PD-1+, BTLA+, Tim-3−. This comprehensive phenotypic study confirms TIGIT together with PD-1 as a discriminatory marker of dysfunctional HCV-specific CD4+ T cells.

## Introduction

In the majority of hepatitis C virus (HCV) infected patients, the virus persists and the patients progress to chronic infection, although a minority of individuals is able to spontaneously control viral replication^1–5^. In addition to the influence of genetic factors like the IL-28 polymorphism that strongly determines the course of natural infection, it is thought that HCV-specific CD4+ T cell dysfunction followed by CD8+ T cell exhaustion and viral escape is the main reason for this loss of viral control^1,6–8^. Co-inhibitory receptors are critical regulators of T cell exhaustion in the context of acute and chronic viral infections. We and others could previously show that PD-1 is a central regulator of T cells in HCV infection^9,10^. Further studies show that exhausted CD8+ T cells in chronic HCV express multiple co-stimulatory and co-inhibitory receptors that converge to keep chronically activated effector CD8+ T cells in check^9–14^. However, less is known about the expression of different co-inhibitory receptors on virus-specific CD4+ T cells mainly due to technical reasons since virus-specific CD4+ T cells have a lower frequency than virus-specific CD8+ T cells in the peripheral blood^1,15^. A recent human study of the co-inhibitory receptor distribution for peptide-stimulated HIV-specific CD8+ as well as CD4+ T cells showed a varying phenotypic and functional pattern according to the disease status of the HIV infection and subset analysed^16^.

In tumour and chronic infection mouse models, the essential role of TIGIT (T cell immunoglobulin and immunoreceptor tyrosine-based inhibitory motif [ITIM] domain) as a regulator of the anti-tumour and anti-viral CD8+ T cell response has been demonstrated^17–20^.

Recently, is has been shown that increased frequencies of TIGIT+ and TIGIT+ PD-1+ CD8+ T cells correlated with parameters of HIV disease progression^21^. *Ex vivo* combinational antibody blockade of TIGIT and PD-L1 enhanced anti-tumour immunity^18,22^, restored viral-specific CD8+ T cells, and reinvigorated the CD4+ T cell response^17,21^.

To assess the pattern of immune-modulatory receptor expression of virus-specific CD4+ T cells in viral hepatitis in more detail^11,23–30^, we analysed the co-inhibitory receptor TIGIT together with an array of different co-inhibitory molecules like PD-1, BTLA, Tim-3 as well as OX40 and CD226 (DNAM-1) on *ex vivo* bulk and MHC class II tetramer+ HCV-specific T cells^24,31–40^. In parallel, we stained a subset of patients with HCV-specific MHC class I tetramers to compare co-inhibitory molecule expression pattern of HCV-specific CD4+ versus HCV-specific CD8+ T cells.

Here, we present a comprehensive analysis of the expression pattern of TIGIT of HCV-specific CD4+ T cells as part of a network of co-inhibitory and co-stimulatory receptors that are known to induce T cell dysfunction and which is possibly associated with loss of viral control in the majority of acutely HCV infected patients.

## Results

### Increased expression level of TIGIT on bulk CD4+ T cells of patients with acute and chronic HCV infection

Recently, a critical role for TIGIT in regulating virus-specific CD4+ T cell responses in chronic HIV infection^41^ has been described, but little is known about the role of TIGIT together with PD-1 and TIGIT’s complementary receptor CD226 on T cells of HCV patients. The aim of this study was to comprehensively analyze the expression pattern of TIGIT together with additional co-inhibitory molecules on bulk and HCV-specific CD4+ T cells of HCV patients with different disease status.

First, we assessed the *ex vivo* surface expression of TIGIT on total CD4+ T cells of patients with acute (n = 10), chronic (n = 11), spontaneously resolved (n = 8) HCV infection and healthy controls (n = 10) (Table 1, Suppl. 1A–C). We observed a significantly higher frequency of bulk TIGIT+ CD4+ T cells of patients with HCV infection compared to healthy controls. Bulk CD4+ T cells of acutely infected HCV patients tended to have the highest frequencies followed by chronically infected HCV patients and patients with spontaneously resolved HCV infection (Suppl. 1B). Based on the differentiation markers CD45RO and CCR7, we defined naïve and memory subsets (CCR7−/CD45RO–terminal effector-T_EMRA_; CCR7+/CD45RO–naïve T cells-T_naïve_; CCR7−/CD45RO+–effector memory–T_EM_; CCR7+/CD45RO+–central memory–T_CM_) of bulk CD4+ T cells and assessed the TIGIT expression of each memory subset. We could detect a significant higher TIGIT expression on all CD4+ T cell memory subsets of acutely HCV infected patients while the general level of TIGIT was highest across all patient groups in the effector memory compartment and lowest in the naïve T cell compartment (Suppl. 1C).

**Table 1:** Clinical, virological, and immunological patient characteristics.

Infection status	Age/Sex	HLA-class II	Genotype	Peak VL (IU/ml)	VL (IU/ml)*	Peak ALT (U/l)	ALT (U/l)*	Longitudinal samples	Outcome	Therapy
aHCV 1	68/f	DRB1*01:01, *14:01	1a	/	4000000	/	51	yes	cHCV/SVR	peg-IFN/RBV (24 W)
aHCV 2	42/m	DRB1*14:01, *15:01	1a	70000	70000	558	558	no	cHCV	/
aHCV 3	51/f	DRB1*03:01, *15:01	2b	8000	8000	1548	798	no	Sp. R	/
aHCV 4	54/f	DRB1*01:02,*03:01	3a	4000000	30000000	1273	1084	yes	Sp. R	/
aHCV 5	47/f	DRB1*01:02, *15:01	n.a.	Llod	Llod	63	63	yes	Sp. R	/
aHCV 6	22/f	DRB1*01:01, *03:01	1a	400000	30000	368	40	yes	cHCV/SVR	peg-IFN/RBV (24 W)
aHCV 7	32/m	DRB1*13:02,*15:01	n.a.	70000	70000	1317	91	no	Sp. R	/
aHCV 8	44/m	DRB1*01:01, *03:01	1a	30000000	90000	4316	1762	yes	cHCV/SVR	peg-IFN/RBV (24 W)
aHCV 9	38/f	DRB1*03:01, 15:06	3	2070000	30500	521	115	no	cHCV	
aHCV 10	39/m	DRB1*04:01, *15:01	1a	18040000	140	2387	51	yes	cHCV/SVR	peg-IFN/RBV (24 W)
rHCV 11	38/m	DRB1*11:01, *13:02	n.a.	Llod	Llod	32	22	no	/	/
rHCV 12	36/m	DRB1*01:01, *11:01	n.a.	Llod	Llod	23	23	no	/	/
rHCV 13	36/m	DRB1*07:01,*15:01	n.a.	Llod	Llod	66	43	no	/	/
rHCV 14	48/f	DRB1*01:01,*-	n.a.	Llod	Llod	15	10	no	/	/
rHCV15	51/f	DRB1*01:01,*14:01	n.a.	Llod	Llod	94	48	no	/	/
rHCV 16	26/m	DRB1*11:02, *12:01	n.a.	Llod	Llod	90	86	no	/	/
rHCV 17	44/m	DRB1*01:01, *27:05	n.a.	Llod	Llod	84	20	no	/	/
rHCV 18	68/m	DRB1*08:03, *11:01	n.a.	Llod	Llod	32	30	no	/	/
cHCV 19	65/m	DRB1*11:01,*15:01	1a	1000000	800000	100	100	no	/	/
cHCV 20	60/f	DRB1*04:01, *15:01	1b	9000000	9000000	68	54	no	/	/
cHCV 21	28/f	DRB1*13:01, *15:01	1b	2000000	2000000	43	43	yes	SVR	Ledipasvir/Sofusbuvir (12 W)
cHCV 22	56/m	DRB1*11:02, *15:01	1a	30000	10000	374	75	no	/	/
cHCV 23	63/f	DRB1*07:01, *15:01	1b	10000000	2000000	138	54	yes	SVR	Ledipasvir/Sofosbuvir (12 W)
cHCV 24	43/m	DRB1*04:04,*15:01	1b	6000000	5230000	208	128	yes	SVR	Ombitasvir/Paritaprevir/Ritonavir+Dasabuvir (12 W)
cHCV 25	43/f	DRB1*13:01, *15:03	1a	8741517	2884373	43	43	no	/	/
cHCV 26	55/m	DRB1*15:01, *13:01	3a	2775	2775	91	65	no	/	/
cHCV 27	40/f	DRB1*03:01,*15:01	3	4330000	4330000	42	30	no	/	/
cHCV 28	39/f	DRB1*04:08, *15:02	3	24300000	24300000	71	50	no	/	/
cHCV 29	46/m	DRB1*07:01, *15:01	3	15900000	13300000	208	127	yes	SVR	Sofusbuvir/Velpatasvir (12 W)
tHCV 30	62/m	DRB1*01:01	n.a.	Llod	Llod	58	58	no	SVR	peg-IFN/RBV (24 W)
tHCV 31	37/m	DRB1*01:01, *07:01	3	25100000	26	319	43	no	SVR	Sofusbuvir/Velpatasvir (12 W)
tHCV 32	37/m	DRB1*15:01, -	3	17800000	Llod	25	25	no	SVR	Sofusbuvir/Velpatasvir (12 W)
tHCV 33	52/m	DRB1*01:01, *11:01	1a	2000000	Llod	272	107	no	SVR	peg-IFN/RBV (48 W)
tHCV 34	39/m	DRB1*07:01, *11:01	3	850000	Llod	157	25	no	SVR	Sofusbuvir/Velpatasvir (12 W)

### TIGIT, PD-1, BTLA and Tim-3 expression pattern of HCV-specific CD4+ T cells of acutely and chronically infected patients

In order to analyze TIGIT expression of HCV-specific CD4+ T cells, we stained PBMC *ex vivo* with a multicolour FACS panel of patients with acute (n = 10), chronic (n = 11) and spontaneously resolved (n = 8) HCV infection using HLA-DRB1*01:01, DRB1*04:01, DRB1*11:01 and DRB1*15:01-restricted tetramers (Table 2) after bead enrichment as previously described^42^. In accordance with previous reports, the *ex vivo* frequency of HCV-specific CD4+ T cells was highest in patients with acute HCV infection (ranging from 0,005%-0,15%; median 0,05%) followed by patients with spontaneously resolved HCV infection (ranging from 0,0003%-0,1%; median 0,005%) and extremely low in patients with chronic HCV infection (ranging from 0%-0,005%; median 0,0005%) (Suppl. 2A,B).

**Table 2:** HLA multimeric complex information.

HLA-A molecule	HCV Protein	Position	Sequence
**Class II**			
DRB1*01:01	NS4B	aa 1806–1818	TLLFNILGGWVAA
DRB1*04:01	NS3	aa 1248–1262	GYKVLVLNPSVAATL
DRB1*04:01	NS4	aa 1770–1790	SGIQYLAGLSTLPGNPAIASL
DRB1*15:01	NS3	aa 1411–1425	GINAVAYYRGLDVSV
DRB1*15:01	NS3	aa 1582–1597	NFPYLVAYQATVCARA
DRB1*11:01	NS4	aa 1773–1790	QYLAGLSTLPGNPAIASL
**Class I**			
A*02:01	NS3	aa 1073–1081	CINGVCWTV
A*02:01	NS3	aa 1406–1415	KLVALGINAV
A*24:02	E2	aa 717–725	EYVLLLFLL

Information on the MHC class II and I tetramer specificities employed in this study.

Differentiation markers CD45RO and CCR7 were used to define naïve and memory subset (CCR7−/CD45RO–terminal effector-T_EMRA_; CCR7+/CD45RO–naïve T cells-T_naïve_; CCR7−/CD45RO+–effector memory–T_EM_; CCR7+/CD45RO+–central memory**-**T_CM_) of HCV–specific MHC class II tetramer+ CD4+ T cells. The vast majority of HCV-specific CD4+ T cells showed a T_EM_ phenotype independent of the infection stage (Suppl. 3A,B), and there was only a minimal decrease of T_EM_ cells of HCV-specific MHC class II tetramer+ CD4+ T cells of chronically infected HCV patients compared to spontaneously resolved HCV patients.

**Figure 1:**
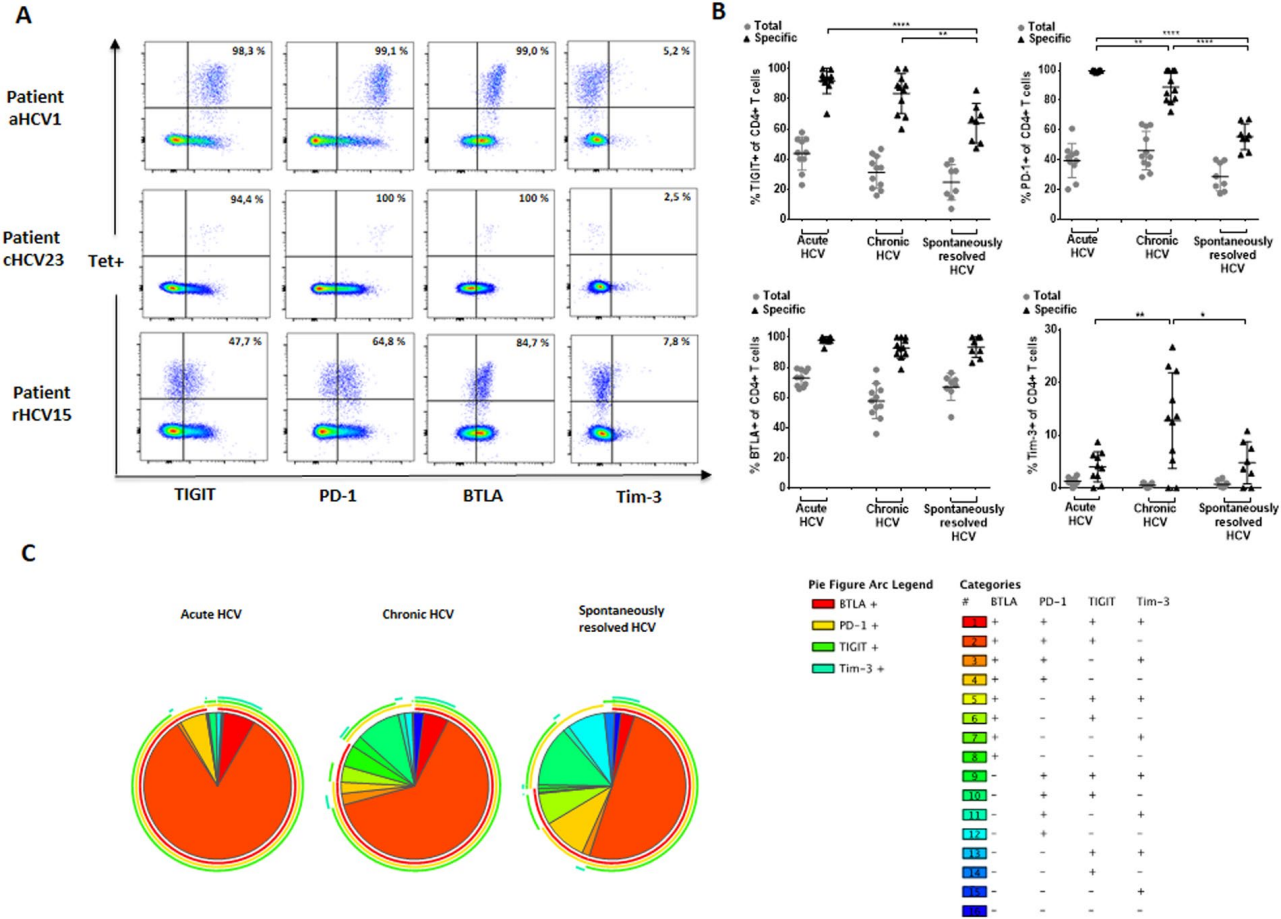
(**A**–**C**) Higher *ex vivo* expression of TIGIT and different co-inhibitory molecules on virus-specific CD4+ T cells of acutely and chronically infected HCV patients compared to patients with spontaneously resolved HCV. (**A**) Representative dot plots depicting the *ex vivo* co-inhibitory receptor expression (TIGIT, PD-1, BTLA, TIM-3) of HCV-specific MHC class II tetramer+ CD4+ T cells of patients with acute, chronic and resolved HCV infection. (**B**) Frequencies of the inhibitory receptors TIGIT, PD-1, BTLA, and Tim-3 on total and HCV-specific CD4+ T cells of patients with acute, chronic, and spontaneously resolved HCV infection. P-values were calculated by the Tukey’s multiple comparison test. P-values smaller than 0.05 were considered significant, where *^,^** and *** indicate p-values between 0.01 to 0.05, 0.001 to 0.01 and 0.0001 to 0.001 respectively. (**C**) SPICE analysis of TIGIT, PD-1, BTLA, and Tim-3 co-expression pattern of HCV-specific CD4+ T cells of patients with acute (n = 10), chronic (n = 10), and spontaneously resolved (n = 8) HCV infection.

Next, we looked at the expression pattern of TIGIT in combination with a number of additional co-inhibitory markers of HCV–specific MHC class II tetramer+ CD4+ T cells of HCV patients with different disease status (Fig. 1A–C). Figure 1A depicts exemplary plots of the inhibitory expression level of TIGIT, PD-1, BTLA, and Tim-3 of MHC class II tetramer+ HCV–specific CD4+ T cells of a patient with a) acute (upper panel), b) chronic (middle panel) and c) spontaneously resolved (lower panel) HCV infection. An increased inhibitory receptor expression level of TIGIT, PD-1, and BTLA, but not Tim-3 was detectable on HCV–specific CD4+ T cells of patients with acute infection and chronic HCV infection. TIGIT and PD-1 expression levels showed marked differences on MHC class II tetramer+ HCV-specific CD4+ T cells between the acute and chronic phase (high expression) and spontaneous resolution (lower expression). BTLA expression levels were generally high in all three patient groups while the Tim-3 expression level of HCV-specific CD4+ T cells was generally much lower – yet highest in chronic HCV patients compared to MHC class II tetramer+ HCV-specific T cells of acutely infected HCV patients or spontaneous resolvers (Fig. 1B). In addition to the differences of the expression frequencies of the different inhibitory molecules at different HCV disease stages, we also observed an significantly increased TIGIT and PD-1 MFI on HCV specific CD4+ T cells from patients with acute (TIGIT; acute vs. resolved: p < 0,0001) (PD-1; acute vs. resolved: p < 0,0001) and chronic (TIGIT; chronic vs. resolved: p < 0,0001) (PD-1; chronic vs. resolved: p < 0,0001) HCV infection compared to patients with spontaneously resolved HCV infection (Suppl. 4A,B). In contrast, while we could not detect any difference in the frequency of BTLA on HCV specific CD4+ T cells between the HCV patients with different disease status, the BTLA MFI was significantly higher on HCV-specific CD4+ T cells from patient with acute HCV infection compared to chronic (acute vs. chronic: p < 0,0009) and spontaneously resolved (acute vs. resolved: p < 0,0001) HCV infection (Suppl. 4C).

In order to define the detailed expression signature of different co-inhibitory molecules on HCV-specific CD4+ T cells, SPICE analysis^43^ on HCV-specific MHC class II tetramer+ CD4+ T cells of patients with different HCV disease status was performed analysing TIGIT, PD-1, BTLA, and Tim-3 co-expression. This analysis revealed that acutely and chronically HCV infected patients generally showed higher frequencies of HCV-specific CD4+ T cells that expressed three inhibitory receptors (TIGIT+ PD-1, BTLA+, Tim-3−) compared to HCV-specific CD4+ T cells of spontaneously resolved HCV patients (Fig. 1C).

**Figure 2:**
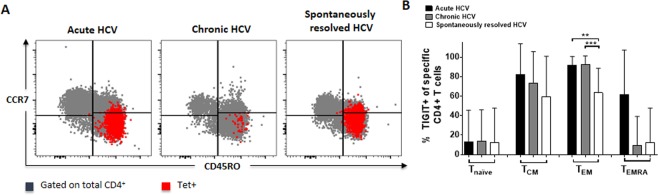
(**A,B**) Memory subset distribution of TIGIT+, HCV-specific CD4+ T cells of HCV patients with acute, chronic and spontaneously resolved infection. The differentiation markers CD45RO and CCR7 were used to analyse the *ex vivo* expression of TIGIT+ HCV-specific MHC class II tetramer+ CD4+ T cells within different memory T cell subsets. (**A**) Representative large dot plots depicting the memory subset distribution of TIGIT+ HCV-specific MHC class II tetramer+ CD4+ T cells (red) on an overlay of gated total CD4+ cells (grey) of patients with acute, chronic and spontaneously resolved HCV infection; memory subset definition: CCR7−/CD45RO–terminal effector T cells-T_EMRA_; CCR7+/CD45RO–naïve T cells-T_naïve_; CCR7−/CD45RO+–effector memory–T_EM_; CCR7+/CD45RO+–central memory–T_CM_. (**B**) Comparison of the TIGIT receptor expression of HCV-specific MHC class II tetramer+ CD4+ T cells and different memory T cell subsets in patients with acute (n = 10), chronic (n = 11), and spontaneously resolved (n = 8) infection. P values were calculated by the Tukey’s multiple comparison test. P-values smaller than 0.05 were considered significant, where *^,^** and *** indicate p-values between 0.01 to 0.05, 0.001 to 0.01 and 0.0001 to 0.001 respectively.

Furthermore, the pattern of TIGIT expression of the different memory subpopulations of MHC class II tetramer+ HCV-specific CD4+ T cells of HCV patients during different stages of disease (Fig. 2A,B) was compared. Recently, it could be shown that TIGIT is mainly expressed on intermediate/transitional and effector T cells of virus-specific CD8+ T cells of patients with HIV^21^. In contrast, here we could detect that TIGIT was significantly higher expressed within the T_EM_ subset of patients with acute and chronic HCV infection compared to patients with spontaneously resolved HCV infection (acute vs. resolved: p = 0,0015) (chronic vs. resolved: p = 0,0009) (Fig. 2B). In the T_CM_ and T_EMRA_ subpopulation, TIGIT expression was relatively stable with no statistically significant difference between patients with acute, chronic or spontaneously resolved HCV infection (Fig. 2B).

### High co-expression of TIGIT and PD-1 on HCV-specific MHC class II tetramer+ CD4+ T cells of patients during acute and chronic HCV infection

**Figure 3:**
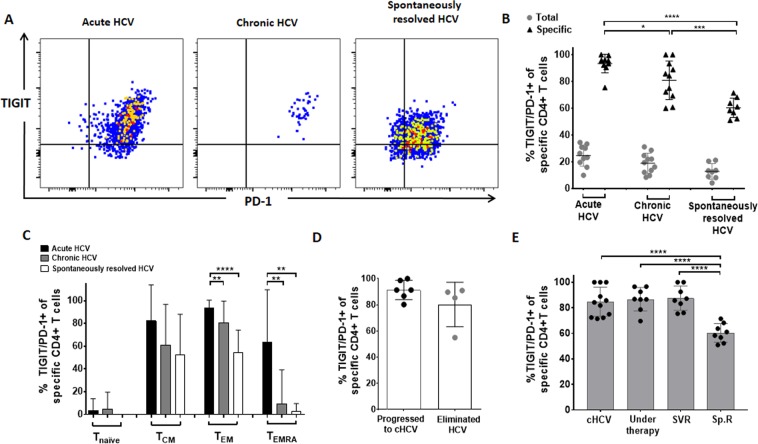
(**A**–**E**) *Ex vivo* TIGIT/PD-1 co-expression of HCV–specific CD4+ T cells of patients with acute, chronic and spontaneously resolved HCV infection. (**A**) Representative large dot plots depicting the *ex vivo* inhibitory molecule co-expression of TIGIT/PD-1 of HCV-specific MHC class II tetramer+ CD4+ T cells of patients with acute, chronic and spontaneously resolved HCV infection. (**B**) TIGIT/PD-1 co-expression on total and HCV-specific CD4+ T cells (**C**) TIGIT/PD-1 co-expression of HCV-specific CD4+ T cells sub-stratified for different memory T cell subsets of patients with acute (n = 10), chronic (n = 11) and spontaneously resolved (n = 8) HCV infection. (**D**) Comparison of TIGIT/PD-1 co-expression of HCV-specific CD4+ T cells during acute infection and between patients who later on progressed to chronic versus patients who spontaneously resolved the HCV infection (**E**) Comparison of the TIGIT/PD-1 co-expression HCV-specific MHC class II tetramer+ CD4+ T cells of patients with chronic HCV (cHCV–without therapy); chronic HCV patients during HCV treatment (Under therapy; 3 patients received a peg-interferon-based and 5 patients a DAA-based therapy); HCV treated chronic patients with sustained virologic response (SVR; 4 patients received a peg-interferon-based and 4 patients a DAA-based therapy); and patients with spontaneously resolved HCV infection (Sp.R). P values were calculated by the Tukey’s multiple comparison test. P-values smaller than 0.05 were considered significant, where *^,^** and *** indicate p-values between 0.01 to 0.05, 0.001 to 0.01 and 0.0001 to 0.001 respectively.

Previous studies have described that especially dual blockade of TIGIT and PD-1 restored anti-viral and anti-tumour T cell effector function in the mouse model^17^, and this led also to an invigoration of the CD8+ T cell function in HIV patients^21^. Therefore, we also specifically evaluated TIGIT/PD-1 co-expression of total and HCV-specific CD4+ T cells of patients with acute, chronic and spontaneously resolved HCV infection (Fig. 3A–C). TIGIT/PD-1 co-expression was significantly increased on HCV-specific CD4+ T cells of acutely and chronically HCV infected patients compared to the expression of patients with spontaneously resolved HCV infection (acute vs. resolved: p < 0,0001) (chronic vs. resolved: p < 0,0001) (Fig. 3B). We further compared the TIGIT/PD-1 co-expression of HCV-specific CD4+ T cell subsets (Fig. 3C). T_EM_ CD4+ T cells of patients with spontaneously resolved HCV infection showed a significantly lower TIGIT/PD-1 co-expression than MHC class II tetramer+ HCV-specific T_EM_ CD4+ T cells of patients with acute (acute vs. resolved: p < 0,0001) or than T_EM_ CD4+ T cells of patients with chronic infection (chronic vs. resolved: p = 0,0044).

Next, we investigated whether there was any difference of the TIGIT/PD-1 co-expression of HCV–specific MHC class II tetramer+ CD4+ T cells during acute HCV infection between patients who progressed to chronic versus patients who spontaneously resolved the infection. During the acute HCV infection we found a slight trend of a reduction of the TIGIT/PD-1 frequency of HCV–specific CD4+ T cells of the patient group that developed a chronic HCV (n = 6) infection compared to patients who later spontaneously eliminated the virus (n = 4) (Fig. 3D), however this difference did not reach statistical significance (p = 0,0933) probably due to the small cohort size and larger cohorts are needed to investigate this question.

We next wanted to explore, whether there is any difference of the TIGIT/PD-1 expression level of HCV–specific CD4+ T cells during and after successful HCV therapy in patients with a sustained virologic response (SVR). Therefore, we compared the TIGIT/PD-1 frequency on MHC class II tetramer+ HCV–specific CD4+ T cells of a subset of chronic HCV patients who underwent HCV therapy. PBMC samples were taken before (cHCV), during and after successful therapy with sustained virologic response (SVR24) (Fig. 3E). Interestingly, we could not detect any difference in TIGIT/PD-1 frequency between a) chronically infected, b) during treatment (cHCV vs. under therapy: p = 0,9692) or c) patients with SVR24 (cHCV vs. SVR: p = 0,9223). This pattern was not different in DAA (direct-acting agents) or PEG-interferon treated patients (data not shown). In contrast, the TIGIT/PD-1 co-expression was significantly lower on HCV–specific CD4+ T cells of patients with spontaneously resolved HCV (under therapy vs. Sp.R: p = <0,0001, SVR vs. Sp.R: p = <0,0001).

### Decreased expression of the co-stimulatory molecule CD226 on HCV-specific CD4+ T cells of chronically infected HCV patients compared to patients with spontaneously resolved HCV

**Figure 4:**
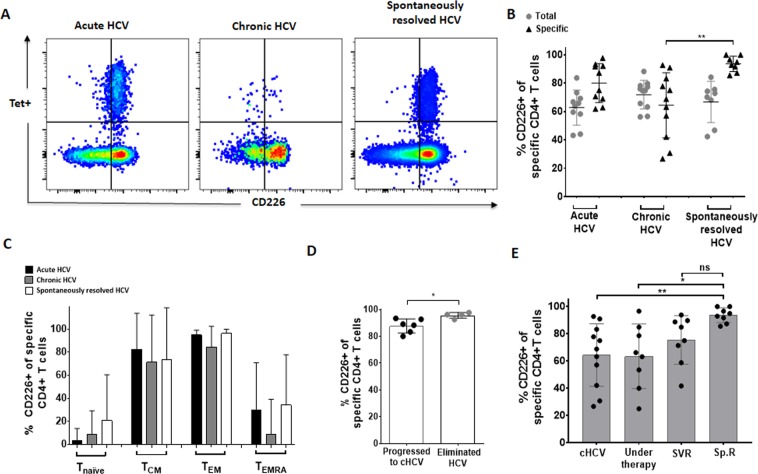
(**A–E**) Lower *ex vivo* expression of the co-stimulatory molecule CD226 on HCV-specific CD4+ T cells of chronically infected HCV patients compared to patients with SVR. (**A**) Representative large dot plots depicting the *ex vivo* co-expression of TIGIT/PD-1 of HCV-specific MHC class II tetramer+ CD4+ T cells of patients with acute, chronic and spontaneously resolved HCV infection. (**B**) CD226 expression of bulk and HCV-specific CD4+ T cells and (**C**) and in different memory subsets of patients with acute (n = 10), chronic (n = 11) and spontaneously resolved (n = 8) HCV infection. (**D**) Comparison of the CD226 expression of HCV-specific CD4+ T cells during the acute HCV phase between patients who later progressed to chronic infection versus patients who spontaneously resolved the HCV infection (**E**) Comparison of the CD226 expression HCV-specific MHC class II tetramer+ CD4+ T cells of patients with chronic HCV (cHCV–without therapy); chronic HCV patients during HCV treatment (Under therapy; 3 patients received a peg-interferon-based and 5 patients a DAA-based therapy); HCV treated chronic patients with sustained virologic response (SVR; 4 patients received a peg-interferon-based and 4 patients a DAA-based therapy); and patients with spontaneously resolved HCV infection (Sp.R). P values were calculated by tukey’s multiple comparison test. P-values smaller than 0.05 were considered significant, where *^,^** and *** indicate p-values between 0.01 to 0.05, 0.001 to 0.01 and 0.0001 to 0.001 respectively.

In a next step we took a closer look at the expression level of the complementary stimulatory receptor of TIGIT: DNAX accessory molecule 1 (DNAM-1; also called CD226) expression of HCV–specific MHC class II tetramer+ CD4+ T cells at different stages of HCV infection (Fig. 4A–E). Previously it has been suggested that upregulation of TIGIT can potentially inhibit other co-stimulatory molecules like CD226^17,44^. Generally, CD226 was expressed on the majority of HCV-specific MHC class II tetramer+ CD4+ T cells (Fig. 4A). However, there were certain differences according to the T cell and HCV disease status (Fig. 4B) and memory T cell subset (Fig. 4C): CD226 expression was significantly lower on HCV-specific CD4+ T cells of patients with chronic HCV infection compared to patients with spontaneously resolved HCV infection (chronic vs. resolved: p = 0,0010). In our cohort, there were 6 patients with acute HCV infection who went on to become chronically infected and 4 patients who spontaneously resolved the acute HCV infection. Of note, the initial CD226 expression was slightly, but significantly lower in patients who had a chronically evolving disease course (Fig. 4D). Of particular interest, we also analysed CD226 expression of HCV-specific T cells before, during and after successful antiviral therapy. There was an increase of the CD226 expression of HCV-specific CD4+ T cells after HCV therapy initiation and patients with SVR showed comparably high levels to patients with spontaneously resolved HCV infection (Fig. 4E). This pattern did not differ between in DAA versus PEG-interferon treated patients (data not shown).

### TIGIT MFI on HCV-specific CD4+ T cells decreased after HCV-therapy initiation in chronic HCV infection

One major finding of the current study is that the frequency of TIGIT alone or together with PD-1 on HCV–specific CD4+ T cells of patients with chronic HCV infected who were treated remains high, -even after successful therapy and resulting SVR. Nevertheless, we further determined the inhibitory expression level of MHC class II tetramer+ HCV-specific CD4+ T cells longitudinally of 4 chronic HCV infected patients who received DAA therapy. We could not detect any difference of the inhibitory expression level percentage-wise at any time point after DAA initiation (data not shown). However, we observed a large and significant drop of the PD-1 and TIGIT mean fluorescence intensity (MFI) on HCV–specific CD4+ T cells of all patients after DAA therapy initiation (Suppl. 4A–C). Furthermore, we investigated the MFI of TIGIT, PD-1, and CD226 on HCV–specific MHC class II tetramer+ CD4+ T cells in longitudinal samples from the acute patients aHCV1 and aHCV6 who later progressed to chronic infection and were eventually antivirally treated with PEG-IFN during the acute phase, as well as of patient aHCV4 who spontaneously eliminated the virus (Suppl. 5A,B). As expected, we could detect a decrease of the PD-1 MFI after HCV therapy initiation while the TIGIT MFI slightly decreased. In patient aHCV4 who spontaneously resolved the acute HCV infection, an even more profound parallel drop of PD-1 and TIGIT could be observed (Suppl. 5A,B).

### HCV–specific MHC class II tetramer+ CD4+ T cells are predominantly TIGIT^high^ in patients with acute and chronic HCV infection

**Figure 5:**
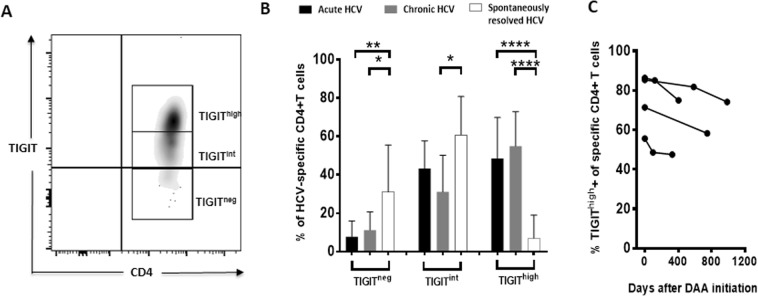
(**A–C**) *Ex vivo* frequency of HCV–specific TIGIT^high^ and TIGIT^int^ CD4+ T cells of HCV patients with acute, chronic and spontaneously resolved infection and longitudinally during DAA therapy. (**A**) Depicting the gating strategy of HCV-specific MHC class II tetramer+ CD4+ T cells divided into subpopulations (TIGIT^high/int/neg^) based on the intensity of TIGIT expression and (**B**) the frequency of TIGIT^high^ and TIGIT^int^ T cells of patients with acute, chronic and spontaneously resolved HCV infection. (**C**) The TIGIT^high^ frequency of HCV–specific CD4+ T cells was analysed in longitudinal samples of chronic HCV patients before, during and after DAA therapy. P values were calculated by Tukey’s multiple comparison test. P-values smaller than 0.05 were considered significant, where *^,^** and *** indicate p-values between 0.01 to 0.05, 0.001 to 0.01 and 0.0001 to 0.001 respectively.

Since it was previously reported that HIV-specific TIGIT^high^ CD4+ T cells (that is TIGIT+ T cells with high MFI) were especially negatively correlated with polyfunctionality and displayed a diminished expression of CD226^45^. We next investigated the MFI of TIGIT expression on CD4+ T cells of HCV patients with different disease stage (Fig. 5A–C). To this aim, we divided the MHC class II tetramer+ HCV-specific CD4+ T cells into subpopulations (hi/int/neg) based on the intensity of TIGIT expression (Fig. 5A). Here, we observed that TIGIT^high^ cells were significantly more frequent in patients with acute and chronic compared to patients with spontaneously resolved HCV infection (acute vs. resolved: p = <0,0001, chronic vs. resolved: p = <0,0001) (Fig. 5B). In contrast, only a small fraction of HCV-specific CD4+ T cells of acutely and chronically infected patients were TIGIT^int^ or TIGIT^neg^^45^. Subsequently, we investigated whether there was any difference in the frequency of TIGIT^high^ CD4+ T cells in longitudinal samples during DAA therapy of four chronic HCV infected patients (Fig. 5C). The frequency of TIGIT^high^ HCV-specific MHC class II tetramer+ CD4+ T cells decreased after DAA initiation in all four patients, but remained at a higher level compared to the TIGIT^high^ frequency of spontaneously resolved patients. The results might indicate that not only the overall frequency of TIGIT cells, but rather the intensity of the expression of this co-inhibitory molecule influences the T cell functionality and a similar observation has been made in HIV infection^45^.

### Definition of the expression pattern of the TIGIT/CD226 axis of HCV-specific CD8+ T cells at different stages of infection

**Figure 6:**
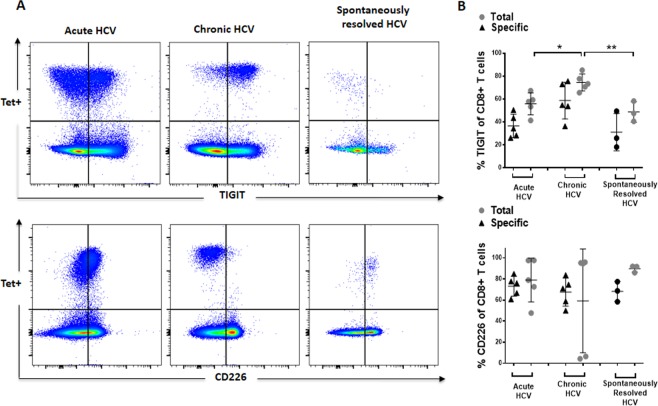
(**A,B**) *Ex vivo* TIGIT and CD226 expression of HCV–specific CD8+ T cells of patients with acute, chronic and spontaneously resolved HCV infection. Representative dot plots depicting the inhibitory receptor expression of HCV-specific MHC class I tetramer+ CD8+ T cells of patients with acute, chronic and spontaneously resolved HCV infection. Comparison of the TIGIT and CD226 frequency of total and HCV-specific CD8+ T cells of HCV patients with acute, chronic and spontaneously resolved HCV infection. P-values were calculated using one-way ANOWA, followed by Tukey’s multiply comparisons test. P-values smaller than 0.05 were considered significant, where *^,^** and *** indicate p-values between 0.01 to 0.05, 0.001 to 0.01 and 0.0001 to 0.001 respectively.

TIGIT has previously been described as an exhaustion marker of antigen-specific CD8+ T cells^18,21^. The co-inhibitory phenotype of MHC class I + II tetramer+ virus–specific CD4+ and CD8+ T cells in HCV infection has rarely been studied side-by-side. We conducted additional preliminary experiments to also understand the expression pattern of our panel of co-inhibitory markers and TIGIT of HCV-specific CD8+ T cells (Fig. 6A–C, Suppl. 6 and data not shown). Of note, the expression pattern differed between HCV-specific MHC class I/II tetramer+ CD8+ and CD4+ T cells: TIGIT expression level of MHC class I HCV-specific CD8+ T cells was generally lower in acute and spontaneously resolved HCV infection but much higher in patients with chronic (chronic vs acute: p = 0,0162, chronic vs resolved: p = 0,0056) HCV infection (Fig. 6A,B). The expression of CD226 on MHC class I tetramer+ HCV-specific CD8+ T cells was variable in chronic HCV infection (Fig. 6C). Three chronically infected patients expressed CD226 at an intermediate level, however two patients showed a complete downregulation of this molecule. Of note, the patients who expressed CD226 at an intermediate level were all HLA-A*02.01 and the other HLA-A*24:02. However, sequence of the circulating virus was not available and viral escape might be a possible explanation for this finding.

## Discussion

Virus-specific CD4+ T cells are primed during acute HCV infection regardless of the outcome but seem to have proliferative defects in patients with chronically evolving disease, and the HCV-specific T cell response wanes further over time^1,6,46–48^. It is generally believed that the reason for this dysfunction is immune exhaustion, a mechanism that has been well-described for CD8+ T cells in murine models and different human chronic viral infections and which is associated with reduced cytokine production, impaired proliferate potential and an upregulation of different co-inhibitory molecules on T cells^31,49^. Recently, Johnston *et al*. (2014) identified a critical role for TIGIT in regulating exhausted CD8+ T cell responses in cancer and chronic infections and additionally identified the TIGIT/CD226 pathway, which provides significant interest for a combined blockade with the PD-1 pathway in order to strengthen the CD8+ T cell response^17^. Indeed, several groups could show that *in vitro* blockade of the TIGIT pathway reinvigorates the exhausted T cell response and first clinical trials in cancer are underway^50^.

In the current study, we studied the co-inhibitory marker expression pattern of CD4+ T cells and could show that TIGIT expression was upregulated on HCV-specific CD4+ T cells of patients with acute and chronic HCV infection compared to patients who spontaneously resolved the HCV infection. The TIGIT expression was closely linked to the co-expression of the inhibitory receptors PD-1 and TIGIT/PD-1 co-expression on HCV-specific CD4+ T cells seemed to be the best combination of co-inhibitory markers to discriminate patients with acute and chronic disease versus spontaneous resolution of HCV, and we observed a significant decrease of TIGIT and PD-1 MFI after antiviral HCV therapy. TIGIT’s complementary receptor CD226 was downregulated on HCV-specific CD4+ T cells in chronically HCV infected patients and of particular interest, slightly increased after antiviral HCV therapy initiation.

Viral clearance of the chronic strain of LCMV in mice by combined blockade of TIGIT and PD-L1 provided the first evidence of the advantages of targeting these two pathways^17^. In addition, targeting TIGIT and PD-L1 on tumour-infiltrating CD8+ T cells in patients with advanced melanoma synergistically improves potent anti-tumour responses^18^. Here, we could detect an elevated PD-1/TIGIT co-expression on HCV-specific CD4+ T cells of acutely and chronically infected patients compared to patients with spontaneously resolved infections. In addition, the PD-1/TIGIT co-expression remained elevated despite antigen removal due to successful antiviral therapy with sustained virologic response (SVR), indicative of only a partial recovery of the HCV-specific T cell response in these patients. Indeed, it is widely known that spontaneously resolved HCV infection partly protects patients of developing a chronic course during a re-infection, which is not the case in re-infected HCV patients with a former SVR by therapy^1,51^.

Furthermore, we investigated whether there is any difference of the TIGIT/PD-1 co-expression during early acute HCV infection between patients who later progressed to either chronic infection versus spontaneous resolution of the HCV infection. We detected a slight statistical trend of a lower TIGIT/PD-1 frequency on HCV–specific CD4+ T cells in the patient group that later resolved the HCV infection. This difference did not reach statistical significance and larger cohorts are needed to investigate whether TIGIT/PD-1 expression could serve as a predictive biomarker combination for clinical outcome. Our phenotypic data would fit the results of previous studies that found that especially high intensity of TIGIT expression together with PD-1 marks dysfunctional T cells^45^.

Intriguingly, Johnston *et al*. found TIGIT’s immunomodulatory effects also depend on CD226 expression^17^. Previous studies have shown a perturbed TIGIT/CD226- axis for virus-specific T cells in HIV infected individuals^45^. Our study aligns well with these previous observations. Here, we observed a reduced CD226 expression level of HCV-specific CD4+ T cells in patients with chronic HCV infection compared to patients with spontaneously resolved HCV infection. The competition for the ligand could partially explain this shifted TIGIT/CD226 axis. In particular, TIGIT’s ability to interfere with CD226 signalling by physically preventing homodimerization represents an important mechanism by which inhibitory receptors can exert their immunomodulatory effects^29,52^. Of note, during acute HCV infection CD226 was decreased on HCV-specific CD4+ T cells of patients who progressed later to chronic HCV infection. This might indicate that the expression of the co-stimulatory receptor CD226 could potentially serve as a prediction marker of spontaneous HCV eradication. Likewise, we observed a slight -but significantly- higher CD226 expression of HCV-specific CD4+ T cells in patients with SVR after antiviral HCV therapy for chronic infection. Recent genome-wide association studies revealed susceptibility variants of polymorphisms in the CD226 gene in distinct autoimmune diseases leading to reduced CD226 cell-surface in distinct T cell subsets^53^. In follow-up studies, we will try to elucidate this complex relationship of CD226 expression with T cell activation and exhaustion.

We also extended the analysis of HCV-specific CD4+ T cells and compared the TIGIT and CD226 expression of HCV-specific CD4+ versus CD8+ T cells of acutely and chronically versus HCV patients with spontaneous resolution of infection. In line with prior studies, we could detect a significant increase of TIGIT expression on HCV-specific CD8+ T cells of patients with chronic HCV infection compared to patients with acute and spontaneously resolved HCV infection. However, it is important to note that the co-inhibitory expression pattern differed somewhat between HCV-specific CD4+ and CD8+ T cells. For example, TIGIT expression on HCV MHC class I tetramer+ CD8+ T cells was low during the acute infection and higher levels were only detectable on HCV-specific CD8+ T cells in chronically infected patients. It is intriguing to speculate whether indeed the virus-specific CD4+ cells exhaust faster than the corresponding HCV-specific CD8+ T cells^1,54,55^.

Our study is one of the most detailed phenotypic immunological studies of co-inhibitory molecule expression of HCV-specific CD4+ in parallel with CD8+ T cells in patients at different stages of HCV using MHC class I + II tetramers to date. However, this study has certain shortcomings inherent of translational cohort studies. We were limited in terms of number of cells, the number of antibodies that could be stained in one individual FACS panel and only basic memory subpopulations were analysed, the patient groups were rather small; and patients were antivirally treated with the different HCV drug regimens (some patients were still treated with interferon-based regimens). Also, patients had different HCV genotypes and sequences of circulating virus were not available and the presence of possible viral escape mutations was not formally tested for the respective epitopic regions and HCV-specific tetramer responses.

In summary, our findings confirm TIGIT together with PD-1 as a potential novel signature marker combination of dysfunctional HCV-specific CD4+ T cells and suggest that TIGIT along with other checkpoint receptors may be a curative target to reverse T cell exhaustion in chronic viral diseases or cancer.

## Material and Methods

### Patient cohort

PBMC of HCV infected patients (n = 39) and of uninfected healthy controls (n = 10) were collected at the University Medical Center Hamburg-Eppendorf and stored in liquid nitrogen (−196 °C). Written informed consent was given by all patients and the study was approved by the local ethics board of the Ärztekammer Hamburg WF14-09, PV4780, PV4081, and all experiments were performed in accordance to relevant guidelines and regulations. Table 1 shows the detailed clinical, virologic and immunological information of 29 patients who were further stratified into 4 groups according to their disease stage^56^ in acute HCV (n = 10), chronic HCV (n = 11), SVR (n = 4) and spontaneously resolved HCV (n = 8) whose PBMC were used for further detailed phenotypic *ex vivo* MHC class I + II tetramer analysis.

### HLA typing

High definition molecular HLA class I and II typing were performed at the Institute of Transfusion Medicine at University Medical Center Hamburg-Eppendorf by polymerase chain reaction-sequence specific oligonucleotide (PCR-SSO) using the commercial kit SSO LabType (One Lambda, Canoga Park, CA, USA)^57^.

### MHC class I and MHC class II tetramer staining and enrichment

HCV-specific MHC class I and II tetramers used in this study are shown in Table II. MHC class I and class II tetramer-associated magnetic bead enrichment technique was performed as previously described^42^. Briefly, cryopreserved PBMC were thawed and stained with PE-labeled HLA class I or class II matching tetramers. Tetramer-enrichment was performed with anti-PE microbeads applying MACS technology (Miltenyi Biotec, Germany) according to the manufacturer’s protocol. Pre-, enriched and depleted Tetramer fractions were further analysed by flow cytometry using BD LSRFortessa™. Frequencies of virus-specific CD8+ and CD4+ T cells were calculated as previously described^42^.

### Multiparametric flow cytometry

For multiparametric flow cytometry analysis, PBMC were stained and enriched with the matched MHC class I or class II tetramer. To exclude dead cells in the subsequent analysis, PBMC were stained with the LIVE/DEAD™ Fixable Near-IR dye (Thermo Fisher, Germany) according to the manufacturer’s protocol. PBMC were stained with appropriate fluorochrome-conjugated surface antibodies, including anti-CD3 (OKT3), anti-CD4 (RPA-T4) anti-CD45RO (UCHL1), anti-CCR7 (G043H7), anti-CD226 (DX11), anti-TIGIT (A15153G), anti-BTLA (MIH26), anti-LIGHT (115520), anti-Ceacam1 (ASL-32), anti-Tim-3 (F38-2E2), anti-PD-1 (EH12.2H7), anti-OX40 (ACT-35), anti-CD14 (63D3), anti-CD19 (HIB19) (from Biolegend, Koblenz or BD Biosciences, Heidelberg, Germany) for 20 min at RT in the dark. After surface staining, cells were washed once with PBS and were then resuspended in 0.5% paraformaldehyde.

### Statistical analysis

All flow cytometric data were analysed using FlowJo version 10.4.2 software (Treestar, Ashland, OR, USA). Statistical analysis was carried out using Prism 6.0 software (GraphPad Software, San Diego, CA). All groups were tested for normal distribution with the Kolmogorov-Smirnov test and were compared by the adequate test. For normally distributed data, parametric tests were applied: for two groups the t-test was used, for more than two groups a ANOVA followed by Tukey’s multiple comparisons test was used. Data that was not normally distributed was tested by the Mann-Whitney test for two unpaired groups, by the Wilcoxon test for paired groups, or Kruskal-Wallis test followed by Dunn’s multiple comparisons test for more than two groups, respectively. P-values smaller than 0.05 were considered significant, where *^,^** and *** indicate p-values between 0.01 to 0.05, 0.001 to 0.01 and 0.0001 to 0.001, respectively. Data are expressed as means with standard deviation, respectively (as indicated in the figure legend). Statistical analysis and display of multicomponent distributions was performed with SPICE v5.1^43^.

## Supplementary information

Supplement 1-6
