# Supplementary material for: HCV-specific CD4+ T cells of patients with acute and chronic HCV infection display high expression of TIGIT and other co-inhibitory molecules
Source: Sci Rep. 2019 Jul 23;9:10624. doi: 10.1038/s41598-019-47024-8 (PMC6650447; doi:10.1038/s41598-019-47024-8)
Supplement: Supplementary file 1 — Supplement 1-6 [file 41598_2019_47024_MOESM1_ESM.pdf]

HCV-specific CD4+ T cells of patients with acute and chronic HCV infection display high expression of TIGIT and other co-inhibitory molecules.

Christin Ackermann, Maike Smits, Robin Woost, Johanna M. Eberhard, Sven Peine, Silke Kummer, Matthias Marget, Thomas Kuntzen, William W. Kwok, Ansgar W. Lohse, Thomas Jacobs, Tobias Boettler and Julian Schulze zur Wiesch

Supplement 1

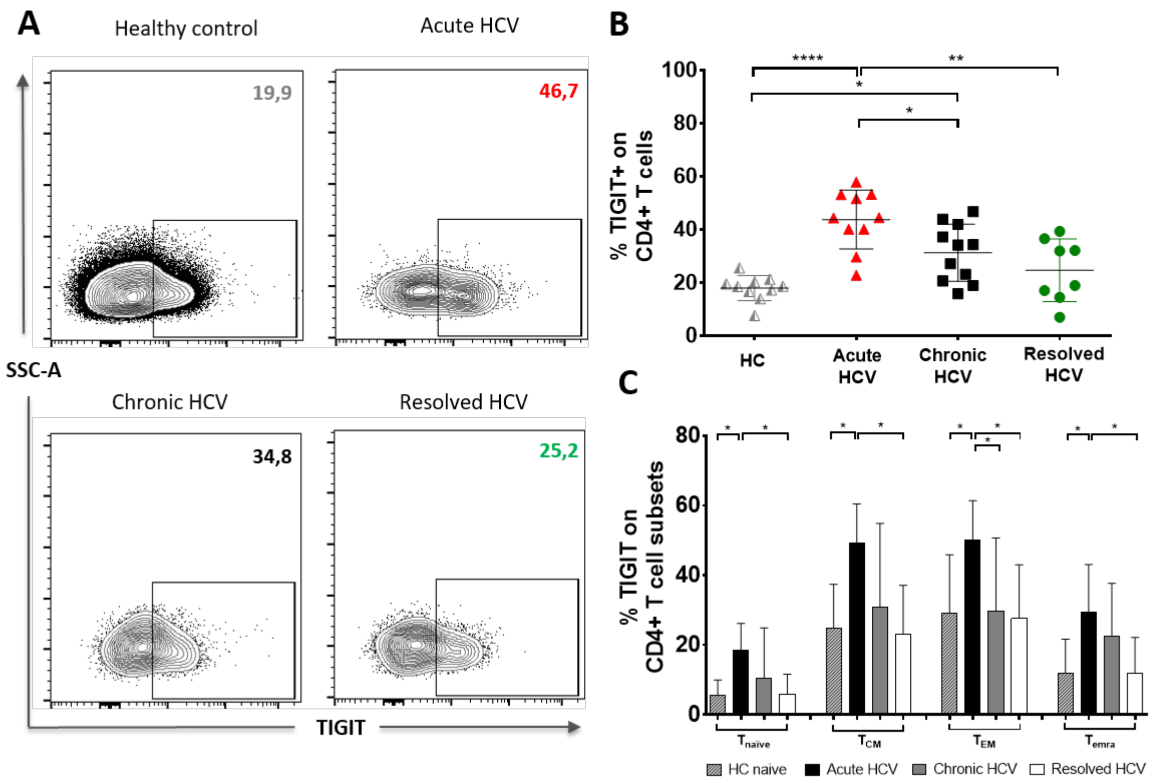

Supplement. 1: **Expression of TIGIT on total CD4+ T cells of HCV patients with different disease status.** All FACS analyses were performed on frozen PBMC samples. (**A**) Representative dot plots depicting the TIGIT expression on total CD4+ T cells and (**B**) the frequency from healthy controls (HC) and patients with acute, chronic and spontaneously resolved HCV infection. (**C**) The differentiation markers CD45RO and CCR7 were used to analyzed the TIGIT expression on naïve and memory CD4+ T cell subsets (CCR7-/CD45RO – terminal effector-T_{EMRA} ; CCR7+/CD45RO – naïve T cells-$T_{naïve}$; CCR7-/CD45RO+ – effector memory – T_{EM}; CCR7+/CD45RO+ – central memory – T_{CM}) from healthy controls and HCV infected patients. P values were calculated using one-way ANOWA, followed by Tukey´s multiply comparisons test. P-values smaller than 0.05 were considered significant, where *, ** and *** indicate p-values between 0.01 to 0.05, 0.001 to 0.01 and 0.0001 to 0.001 respectively.

Supplement 2

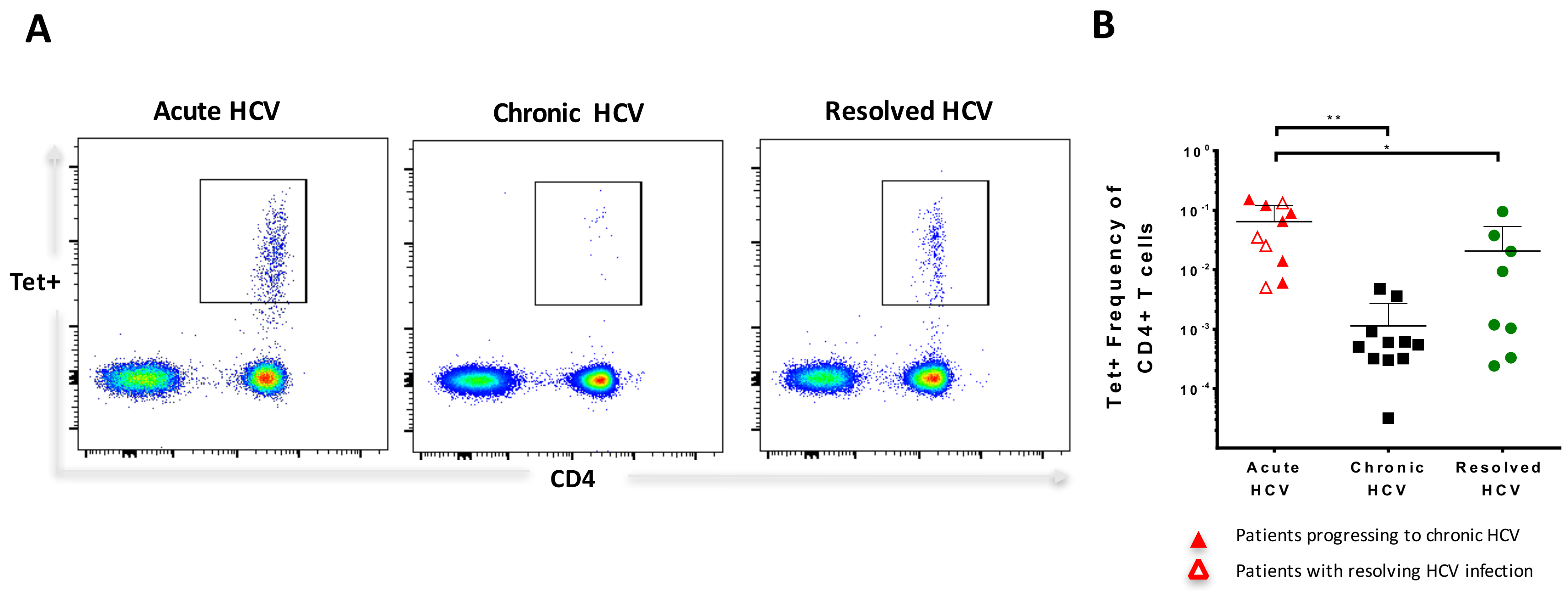

Supplement 2: Frequencies of MHC class II tetramer positive (Tet+) $CD4^+$ T cells of patients with different HCV disease stage. (**A**) Representative dot plots depicting virus-specific T cells, pre-gated on live CD3+ CD4+ lymphocytes from patients with acute, chronic and spontaneously resolved HCV infection. (B) . P values were calculated by tukey's multiple comparison test. P-values smaller than 0.05 were considered significant, where *, ** and *** indicate p-values between 0.01 to 0.05, 0.001 to 0.01 and 0.0001 to 0.001 respectively

Supplement 3

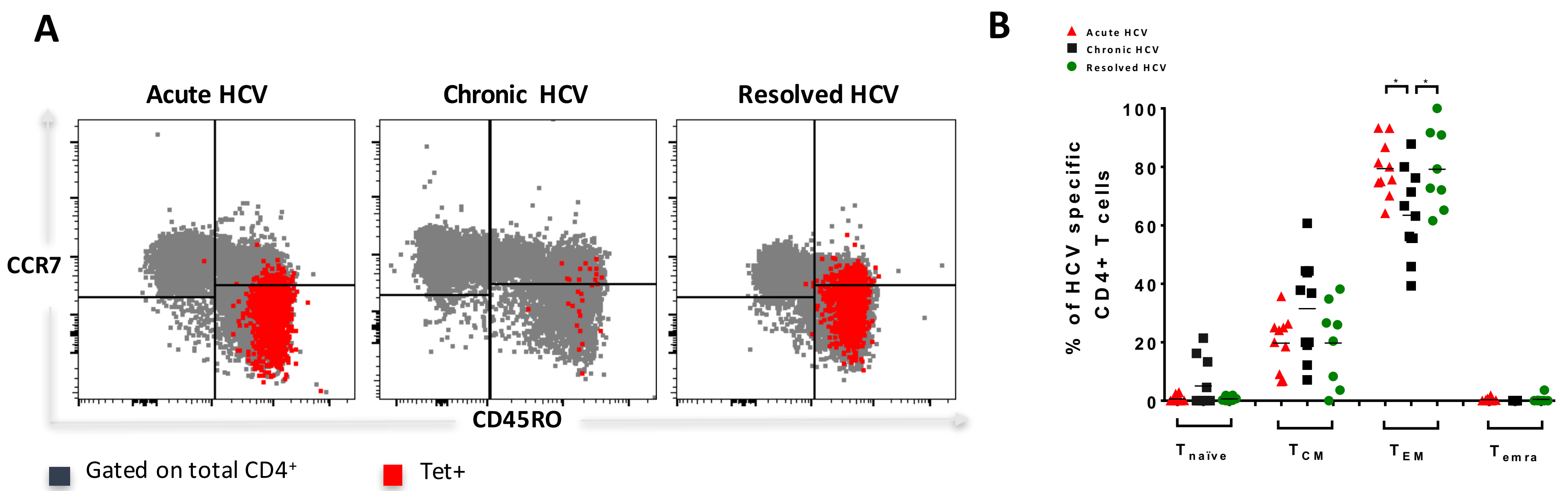

Supplement 3: Differentiation of virus-specific CD4$^+$ T cells of HCV patients with different disease stage. (A) Based on the differentiation markers CD45RO and CCR7 CD4+ T cell were divided in naive and memory subsets (CCR7-/CD45RO – terminal effector-TEMRA ; CCR7+/CD45RO – naïve T cells-Tnaïve ; CCR7-/CD45RO+ – effector memory – TEM; CCR7+/CD45RO+ – central memory – TCM) **.(A)** Representative overlay dot plots show the differentiation of antigen-specific CD4$^+$ T cells and (B) the frequencies of CD4+ T cell subset distribuation from HCV patients with acute, chronic and spontaneously resolved infection. P values were calculated by tukey's multiple comparison test. P-values smaller than 0.05 were considered significant, where *, ** and *** indicate p-values between 0.01 to 0.05, 0.001 to 0.01 and 0.0001 to 0.001 respectively.

Supplement 4

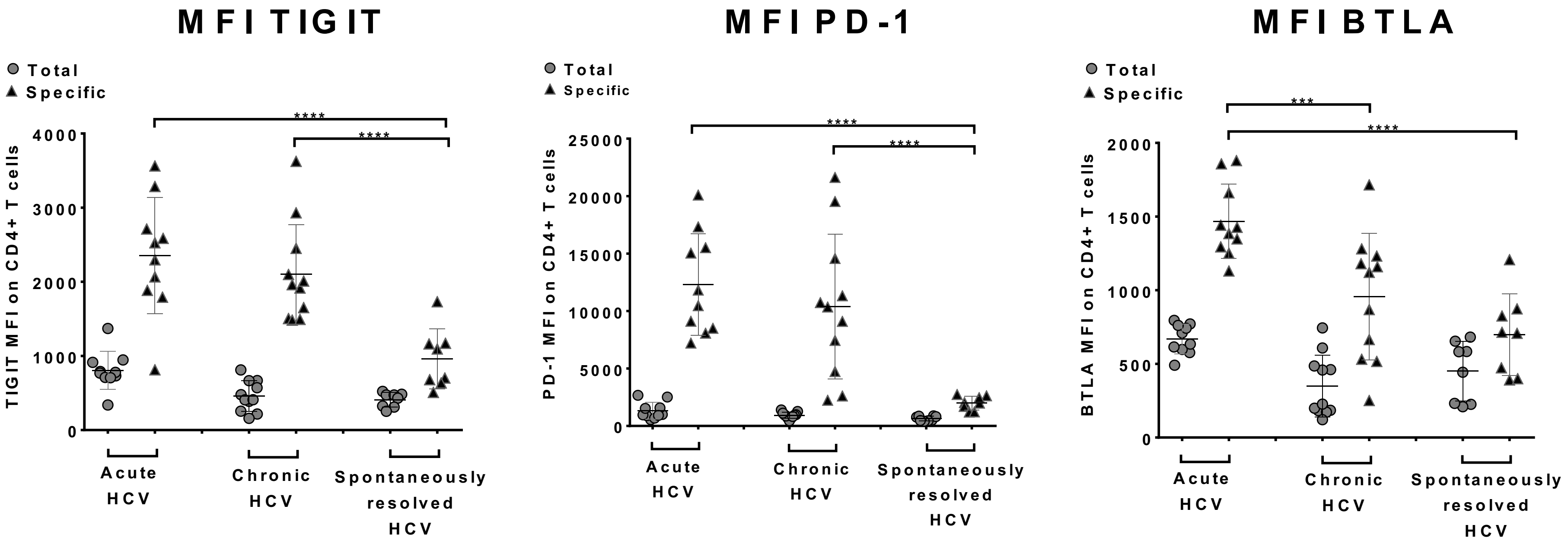

Suppl. 4: Increased MFI of inhibitory markers on virus-specific CD4+ T cells of acutely and chronically infected HCV patients compared to patients with resolved HCV. MFI of Inhibitory receptors (**A**) TIGIT, (**B**) PD-1, and (**C**) BTLA on total and virus-specific CD4+ T cells from patient acute, chronic and resolved HCV infection. P values were calculated by tukey's multiple comparison test. P-values smaller than 0.05 were considered significant, where *, ** and *** indicate p-values between 0.01 to 0.05, 0.001 to 0.01 and 0.0001 to 0.001 respectively.

Supplement 5

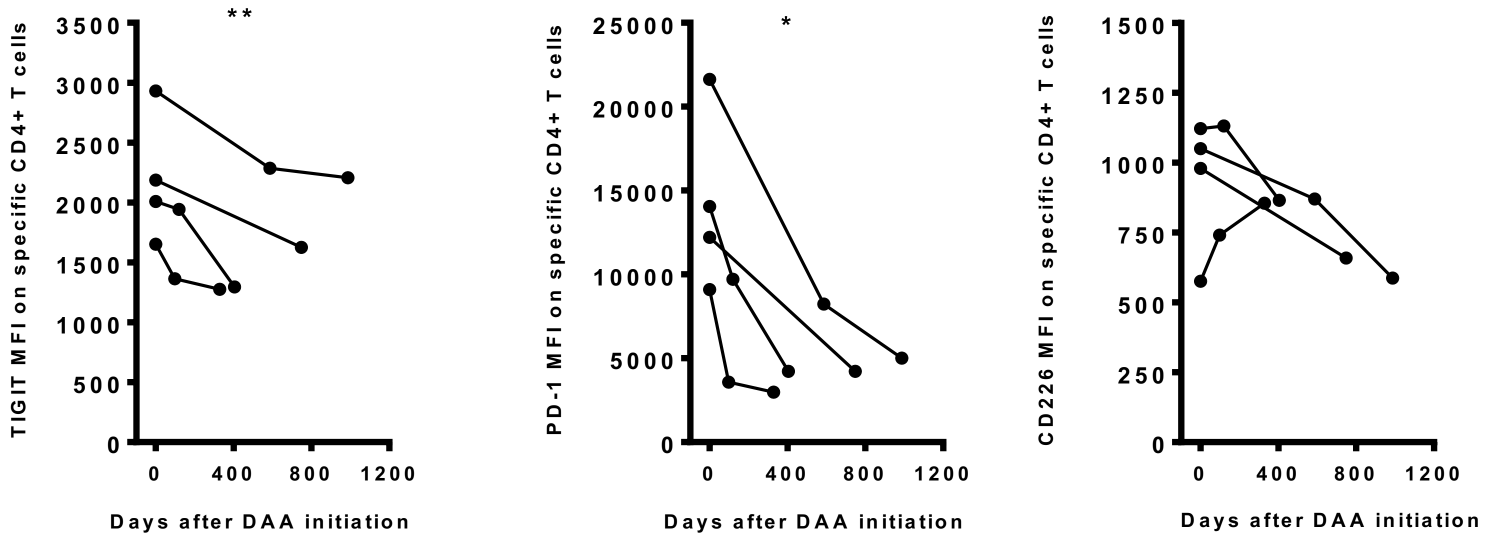

Fig 5. : **Coreceptor MFI during (DAA) therapy.** The MFI of the inhibitory receptors (**A**) TIGIT and (**B**) PD-1 and of the stimulatory receptor (**C**) CD226 were detected in longitudinal samples on virus-specific CD4+ T cell from four chronic HCV infected patients. PBMC samples were taken on day 0 (patients were not yet treated), during and after the end of DAA therapy.

Supplement 6

A **aHCV 6** (Acute – progressed to cHCV and was antivirally treated)

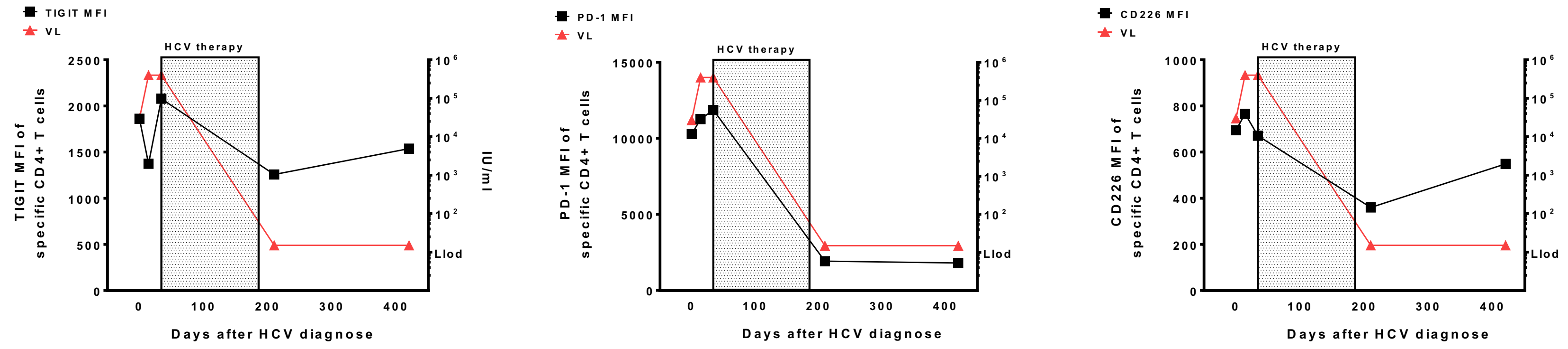

B **aHCV 4** (Acute – spontaneously eliminated the virus)

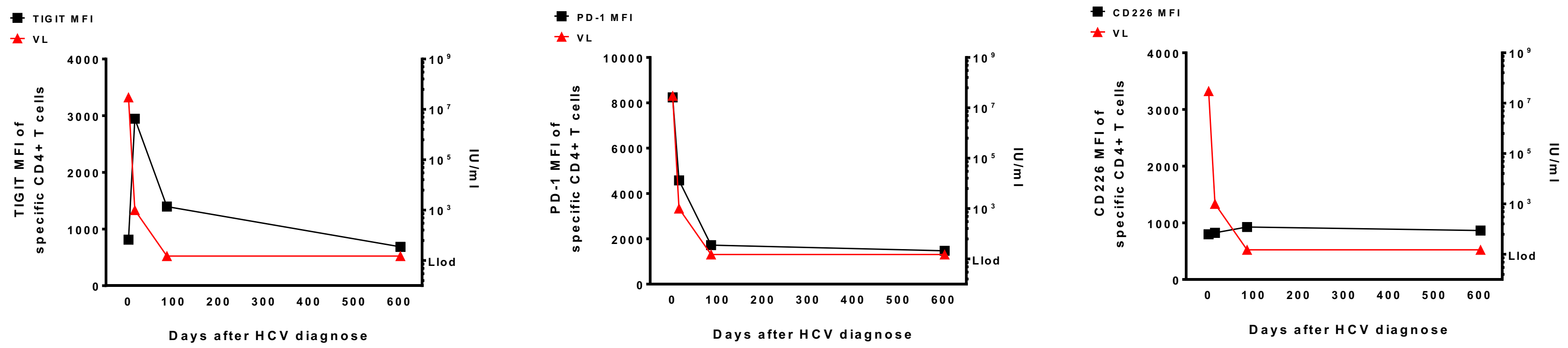

Fig 6. : TIGIT, PD-1 and CD226 MFI was detected in longitudinal samples of HCV infected subjects (A) An HCV patient who was treated during early infection and (B) An HCV patient who spontaneously resolved the infection. PBMC of patient aHCV6 were analyzed during the acute HCV stage, during- and after end of therapy and the MFIs of TIGIT, PD-1 and CD226 are depicted in **6A** together with the corresponding viral load (VL) and duration of the PEG/IFN therapy (24 W). PBMC of patient aHCV4 were analyzed during the acute HCV infection until the spontaneous elimination of the virus the MFIs of TIGIT, PD-1 and CD226 are depicted in **6B** together with the corresponding viral load (VL).
